# Investigation on Insulated, Brain-Implanted Antenna for Highly Reliable Biotelemetry Communication in MICS and ISM Bands

**DOI:** 10.3390/s20010242

**Published:** 2019-12-31

**Authors:** Geonyeong Shin, Ick-Jae Yoon

**Affiliations:** Department of Electrical Engineering, Chungnam National University, Daejeon 34134, Korea; gyshin@cnu.ac.kr

**Keywords:** spherical wave, implanted antenna, small antenna coupling, biotelemetry

## Abstract

We derived a closed-form expression of the maximum power transfer efficiency (MPTE) between a transmitting antenna inside the brain and a receiving antenna outside the head using spherical wave expansion. The derived expression was validated using a FEKO simulation. The properties of the insulator and radiation mode were analyzed in each available medical implant communications service (MICS) and industrial, scientific and medical (ISM) band as a means of increasing the reliability of wireless biotelemetry implementation. Some interesting preceding results in the literature were revisited with the figure-of-merit MPTE. It was also newly found that the effect on MPTE by the physical size and material properties of the insulator in both transverse magnetic (TM) and transverse electric (TE) mode decreases for 2.4 GHz and 5.8 GHz and the loss of the insulator does not have a severe impact on MPTE once the dielectric constant is greater than a certain value. This work can be used as an implanted-antenna design guide for building reliable biotelemetry communication.

## 1. Introduction

Treatment of brain diseases through the analysis of electrical signals from the brain is an interesting research topic. A variety of implantable biotelemetry devices have been developed for this purpose, and methods for the wireless transmission of the detected brain signals are also being actively studied to maximize convenience for patients while avoiding recurrent surgical interventions and the possible risk of infection [1,2,3]. However, the sensor used to detect weak μV–mV brain signals must be located in the lossy brain medium which is difficult for electromagnetic waves to travel through. Moreover, the physical and electrical size of the antenna in such implanted devices must be very small, and this makes the realization of a reliable wireless communication link between the implanted antenna and the external receiver even more challenging.

Despite these difficulties, a number of implanted antennas for biotelemetry systems are currently being studied [4,5,6,7]. To be more specific, they are a multi-layered spiral antenna operating in the MedRadio band (401–406 MHz), an industrial, scientific and medical (ISM) band at 2.4 GHz [4], a planar-inverted F antenna operating in the medical implant communications service (MICS: 402–405 MHz) band, an ISM band at 900 MHz [5], a circular polarized patch antenna operating in the 2.4 GHz ISM band [6] and a microstrip patch antenna operating in the ultra-wideband (UWB: 3.1–10.6 GHz) [7]. The antennas reported in the literature are small enough to be placed in the brain with a good impedance matching characteristic at each medical service band. However, the transmitted signal levels, or radiation power, remain extremely low due to the lossy medium of the brain and the electrically-small size of the antennas. A number of analytic studies have been conducted to learn the effects of lossy medium on the performance of the antennas [8,9,10,11,12,13,14,15]. Due to its small size, the implanted antenna generally radiates the lowest mode only and its radiation characteristic in the presence of a lossy medium, such as one of the human tissues, can be analyzed using spherical wave expansion. This spherical wave analysis can be used not only when the transmitting antenna is located inside the human tissue [8,9,10,11], but when it is located outside as well [12,13]. Such a technique also can be used for studying the radiation characteristics of the antennas immersed in an infinite lossy medium without boundaries [14,15]. For that, the properties of an antenna’s insulating layer, which is essential to protect the device and prevent metal oxidation, were studied undertaken in [8] to examine the poor radiation power of the implanted antennas. It showed how the material property of the insulator influences on the radiated power of the implanted transmitting antenna. One noteworthy finding is that, in the MedRadio band, the total radiated power measured outside the head differs according to the insulator material and the radiation mode of the antenna. This is an interesting result but is limited to MedRadio, although several other frequency bands have been released for medical applications, including the MICS, a particular ISM and UWB [4,5,6,7]. Furthermore, we found that the power transfer ratio between the implanted transmitting antenna and the external receiving antenna of the monitoring system could be more intuitive than the radiated power of the antenna in the brain itself.

In this paper, we study the properties of the insulating layer of the implanted antenna as a means of minimizing path loss to the external antenna of the monitoring system in the MICS and relevant ISM bands. To this end, we derive a closed-form expression of maximum transfer power efficiency (MPTE) between the antennas separated by a multi-layered spherical shell (MSS) to mimic the human head, including the brain. The closed-form expression of MPTE is verified by full-wave EM simulations. Next, the derived solution is used to analyze the effects of different insulator properties on MPTE including: (i) commercially available biocompatible insulators; (ii) variation in the dielectric properties and thickness of the insulator; (iii) the gap size between antenna and insulator; and (iv) the radiation mode. A practical design guide for small implanted antennas with appropriate insulator permittivity and thickness values is also presented. Some preliminary results have been briefly discussed in [16].

## 2. Derivation and Validation of Analytic Solution

### 2.1. The Derivation of an Analytical Soultion

**Figure 1:**
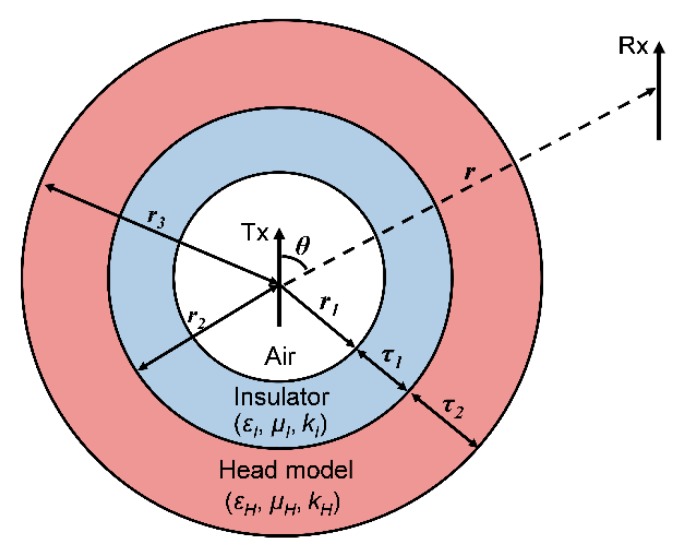
Multi-layered spherical shell (MSS) model for a brain-implanted antenna (Tx) with a receiver antenna (Rx) outside the model.

First, we constructed an MSS consisting of three sections; namely, air, insulator and head (Figure 1), to analyze the influence of the implanted antenna’s insulating layer on MPTE. That MSS configuration was the same as the configuration used in [8]. The head section was filled with a homogeneous material that can satisfactorily replace an inhomogeneous model [12]. The homogeneous, frequency-dependent material properties of the head layer were based on the SAR values reported in [17]. Spherical shell models have been used in deriving analytic solutions for various situations of power loss due to the lossy material with the antenna [8,9,10,11,12,13,14,15]. In particular, it has been shown in [11] that the electric field intensity values of a brain-implanted antenna calculated from an MSS model and from a numerical head phantom are very close. In this work, a transmitting antenna (Tx) surrounded by an insulator was immersed inside a head shell, and the receiving antenna (Rx) was located outside the MSS.

A spherical wave radiator located in an unbounded lossy medium should be isolated from that medium through a lossless layer [14,15]. Otherwise, infinite power must be supplied for a Hertzian dipole to radiate [8]. In order to obtain meaningful results from our analysis of the insulating layer, we located the Tx in a region of lossless air, as depicted in Figure 1. The distance between the Tx and the Rx is *r*, and the position of the Rx with respect to the Tx is expressed as θ. The radius of the air layer is *r*_1_; the radius of the insulator is *r*_2_ with a thickness of *τ*_1_; and the radius of the overall head model is *r*_3_ with a thickness of *τ*_2_, meaning that *r* = *r*_1_ + *τ*_1_ + *τ*_2_. The wave propagation constants for the insulator and the head are $k_{I}=\omega \sqrt{\mu_{I}\varepsilon_{I}}$ and $k_{H}=\omega \sqrt{\mu_{H}\varepsilon_{H}}$, respectively.

To derive MPTE between the Tx and the Rx in the given MSS model, the theoretical upper bound of MPTE between two small antennas in free space in [18] was utilized, given as:

(1) $$MPTE_{fs}=\frac{{\left|X\right|}^{2}}{2-Re\left[X^{2}\right]+\sqrt{4\left(1-Re\left[X^{2}\right]\right)-Im{\left[X^{2}\right]}^{2}}}\;X=\eta_{eff}\cdot T\;T=\frac{3}{2}\cdot \left[-sin^{2\;}\theta \frac{1}{jk_{0}r}+\left(3cos^{2}\;\theta -1\right)\cdot \left(\frac{1}{{\left(jk_{0}r\right)}^{2}}+\frac{1}{{\left(jk_{0}r\right)}^{3}}\right)\right]\cdot e^{-jk_{0}r},$$

where $\eta_{eff}$ is the antenna radiation efficiency, *r* is the distance between the antennas, and $k_{0}=\omega \sqrt{\mu_{0}\varepsilon_{0}}$ is the wave propagation constant in free space. An optimal load to achieve maximum coupling is established at the Rx, and the lowest spherical TM_10_ or TE_10_ radiation mode from an electrically small antenna is assumed. This assumption holds true for the present work because the electrical size of the implanted antenna should be limited to being very small.

To extend MPTE in free space to the MSS model, we first calculate the field intensity from the Tx outside of the MSS. To this end, vector potentials in each region of the MSS are defined. The electric vector potential when the Tx radiates in TE_10_ mode can be defined as:

(2) $$F_{r}=\{\begin{array}{cc} P_{1}(cos\;\theta )\cdot [1\cdot {\hat{H}}_{1}^{\left(2\right)}(k_{0}r)+a_{TE}{\hat{J}}_{1}(k_{0}r)] & \left(r\leq r_{1}\right)\\ P_{1}(cos\;\theta )\cdot [b_{TE}\cdot {\hat{J}}_{1}(k_{I}r)+C_{TE}{\hat{Y}}_{1}(k_{I}r)] & \left(r_{1}\leq r\leq r_{2}\right)\\ P_{1}(cos\;\theta )\cdot [d_{TE}\cdot {\hat{J}}_{1}(k_{H}r)+e_{TE}{\hat{Y}}_{1}(k_{H}r)] & \left(r_{2}\leq r\leq r_{3}\right)\\ P_{1}(cos\;\theta )\cdot f_{TE}{\hat{H}}_{1}^{\left(2\right)}(k_{0}r) & \left(r_{3}\leq r\right) \end{array}$$

where ${\hat{H}}_{n}^{\left(2\right)}\left(x\right)=xh_{n}^{\left(2\right)}\left(x\right)$ is the alternative spherical Hankel function of the second kind, and ${\hat{J}}_{n}\left(x\right)=xj_{n}\left(x\right)$ and ${\hat{Y}}_{n}\left(x\right)=xy_{n}\left(x\right)$ are the alternative, spherical Bessel functions of the first and second kinds [19] (page 460), respectively. We assume $e^{jwt}$ time dependence. Further, *a_TE_* is the amplitude coefficient of the standing wave in the air region ($r\leq r_{1}$); *b_TE_* and *c_TE_* are the standing wave coefficients of the insulator ($r_{1}\leq r\leq r_{2}$); *d_TE_* and *e_TE_* are the coefficients of the standing waves in the head layer ($r_{2}\leq r\leq r_{3}$); and the coefficient *f_TM_* is for the outgoing wave external to the MSS ($r\geq r_{3}$). We set *n* to be 1 since the Tx and the Rx are assumed to radiate only the lowest TE_10_ mode due to their small size. The coefficient values can be determined using the boundary condition of the tangential components of *E_Φ_* and *H_θ_* defined by the *F_r_* at every interface of the region is continuous. In this way, the following matrix can be obtained as:

(3) $$\begin{array}{c} \left[\begin{array}{cccccc} \frac{1}{\varepsilon_{0}}{\hat{J}}_{1}(k_{0}r_{1}) & -\frac{1}{\varepsilon_{I}}{\hat{J}}_{1}(k_{I}r_{1}) & -\frac{1}{\varepsilon_{I}}{\hat{Y}}_{1}(k_{I}r_{1}) & 0 & 0 & 0\\ \frac{k_{0}}{\varepsilon_{0}\mu_{0}}{\hat{J}}_{1}^{\prime }(k_{0}r_{1}) & -\frac{k_{I}}{\varepsilon_{I}\mu_{I}}{\hat{J}}_{1}^{\prime }(k_{I}r_{1}) & -\frac{k_{I}}{\varepsilon_{I}\mu_{I}}{\hat{Y}}_{1}^{\prime }(k_{I}r_{1}) & 0 & 0 & 0\\ 0 & \frac{1}{\varepsilon_{I}}{\hat{J}}_{1}(k_{I}r_{2}) & \frac{1}{\varepsilon_{I}}{\hat{Y}}_{1}(k_{I}r_{2}) & -\frac{1}{\varepsilon_{H}}{\hat{J}}_{1}(k_{H}r_{2}) & -\frac{1}{\varepsilon_{H}}{\hat{Y}}_{1}(k_{H}r_{2}) & 0\\ 0 & \frac{k_{I}}{\varepsilon_{I}\mu_{I}}{\hat{J}}_{1}^{\prime }(k_{I}r_{2}) & \frac{k_{I}}{\varepsilon_{I}\mu_{I}}{\hat{Y}}_{1}^{\prime }(k_{I}r_{2}) & -\frac{k_{H}}{\varepsilon_{H}\mu_{H}}{\hat{J}}_{1}^{\prime }(k_{H}r_{2}) & -\frac{k_{H}}{\varepsilon_{H}\mu_{H}}{\hat{Y}}_{1}^{\prime }(k_{H}r_{2}) & 0\\ 0 & 0 & 0 & \frac{1}{\varepsilon_{H}}{\hat{J}}_{1}(k_{H}r_{3}) & \frac{1}{\varepsilon_{H}}{\hat{Y}}_{1}(k_{H}r_{3}) & -\frac{1}{\varepsilon_{0}}{\hat{H}}_{1}^{\left(2\right)}(k_{0}r_{3})\\ 0 & 0 & 0 & \frac{k_{H}}{\varepsilon_{H}\mu_{H}}{\hat{J}}_{1}^{\prime }(k_{H}r_{3}) & \frac{k_{H}}{\varepsilon_{H}\mu_{H}}{\hat{Y}}_{1}^{\prime }(k_{H}r_{3}) & -\frac{k_{0}}{\varepsilon_{0}\mu_{0}}{\hat{H}}_{1}^{{\left(2\right)}^{\prime }}(k_{0}r_{3}) \end{array}\right]\cdot \left[\begin{array}{c} a_{TE}\\ b_{TE}\\ c_{TE}\\ d_{TE}\\ e_{TE}\\ f_{TE} \end{array}\right]=\\ \left[\begin{array}{c} -\frac{1}{\varepsilon_{0}}{\hat{H}}_{1}^{\left(2\right)}(k_{0}r_{1})\\ -\frac{k_{0}}{\varepsilon_{0}\mu_{0}}{\hat{H}}_{1}^{{\left(2\right)}^{\prime }}(k_{0}r_{1})\\ 0\\ 0\\ 0\\ 0 \end{array}\right] \end{array}$$

Next, to extend MPTE in free space to the MSS model using the obtained coefficients, we define the shell efficiency, $\eta_{shell}$, which shows the ratio between the input power of the Tx and the power radiating out of the shell as:

(4) $$\eta_{shell}=\frac{P_{out}^{shell}}{P_{in}^{shell}},$$

where $P_{in}^{shell}$ is the input power of the Tx in the air region from:

(5) $$P_{in}^{shell}=∯\frac{1}{2}Re\left[{\vec{E}}_{r<r_{1}}\times {\vec{H}}_{r<r_{1}}^{*}\right]\cdot d\vec{S}\;=\frac{4}{3}\pi \eta_{0}\cdot Re\left[j\left\{{\hat{H}}_{1}^{\left(2\right)}\left(k_{0}r\right)+a_{TE}\cdot \hat{J_{1}}\left(k_{0}r\right)\right\}\cdot \left\{{\hat{H}}_{1}^{{\left(2\right)}^{\prime }}{\left(k_{0}r\right)}^{*}+a_{TE}*\cdot {\hat{J}}_{1}^{\prime }{\left(k_{0}r\right)}^{*}\right\}\right],$$

and $P_{out}^{shell}$ is the radiating power of the Tx leaving the shell from:

(6) $$P_{out}^{shell}=∯\frac{1}{2}Re\left[{\vec{E}}_{r_{3}<r}\times {\vec{H}}_{r_{3}<r}^{*}\right]\cdot d\vec{S}\;=\frac{4}{3}\pi \eta_{0}\cdot Re\left[j\cdot \left\{f_{TE}\cdot {\hat{H}}_{1}^{\left(2\right)}\left(k_{0}r\right)\cdot f_{TE}*\cdot {\hat{H}}_{1}^{{\left(2\right)}^{’}}{\left(k_{0}r\right)}^{*}\right\}\right]$$

Ultimately, the MPTE of the MMS model is obtained by multiplying the MPTE in free space by $\eta_{shell}$. The final expression is, thus, given by:

(7) $$MPTE_{shell}=\eta_{shell}\times MPTE_{fs}.$$

MPTE for TM modes can also be derived using duality. It is worth noting that the described derivation procedure follows the work in [20] which solved the case of TM radiation separated by a single shell.

### 2.2. Validation of the Analytical Solution Using a Numerical Simluator

**Figure 2:**
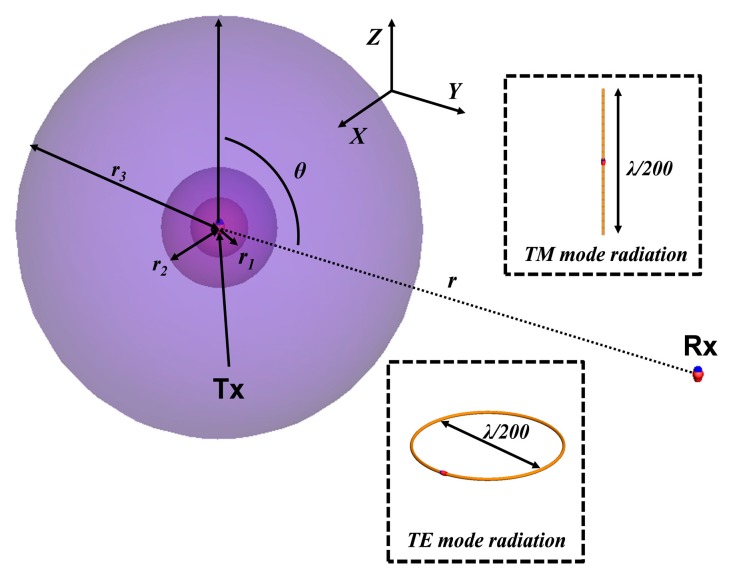
FEKO simulation setup for verification of the derived solution.

We used the numerical simulator FEKO by Altair to verify the derived MPTE expressions. The FEKO simulation uses λ/200-long dipoles for both the Tx and Rx radiating TM_10_ waves and loops with a diameter of λ/200 radiating TE_10_ waves, as shown in Figure 2, where λ is the free space wavelength. A perfect electric conductor was used for the dipoles and loops (i.e., $\eta_{eff}=1$), and an optimal Linville load for maximum coupling of a two-port network was loaded at the Rx [21] (page 476). In the validation, we considered a dielectric material without magnetic loss (*ε_r_*″ ≠ 0; *μ_r_*″ = 0) since the human head is a generally electrically lossy medium with no magnetic loss [22].

**Figure 3:**
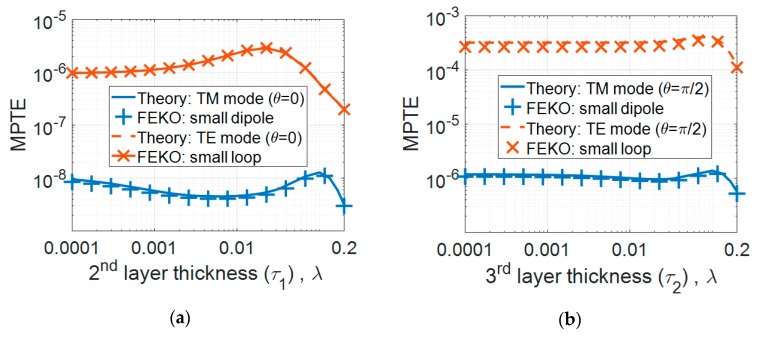
Maximum power transfer efficiency (MPTE) results using the derived theory and FEKO simulation according to (**a**) the thickness of the insulting layer (*τ*_1_) and (**b**) the thickness of the head layer (*τ*_2_).

The validation results are shown in Figure 3. Specifically, Figure 3a presents the MPTE values according to insulator thickness (*τ*_1_) when *r* and *θ* are fixed as 1.0 λ and 0, respectively. The radius of the air-filled region containing the antenna (*r*_1_) was 0.005 λ and the thickness of the head layer (*τ*_2_) was fixed at 0.05 λ, and *τ*_1_ was changed from 0.0001 λ to 0.2 λ. The free-space region was set with material properties of *ε_r_* = 1.0 and *tanδ* = 0.0. The insulating and head layers of the MSS were set with *ε_r_* and *tanδ* values of 5.0 and 1.0 and 10 and 5.0, respectively. The values were arbitrarily chosen in the validation. With *θ* at π/2, the insulator properties were then changed to *ε_r_* = 3.0 and *tanδ* = 0.5, and those of the head layer to *ε_r_* = 5.0 and *tanδ* = 1.0, and the resulting MPTE values are shown in Figure 3b. Then, *τ*_1_ was fixed at 0.05 λ and *τ*_2_ was varied from 0.0001 λ to 0.2 λ. In Figure 3a,b, it can be seen that the MPTE values derived from the proposed solution and from the numerical simulations show good agreement.

**Figure A1:**
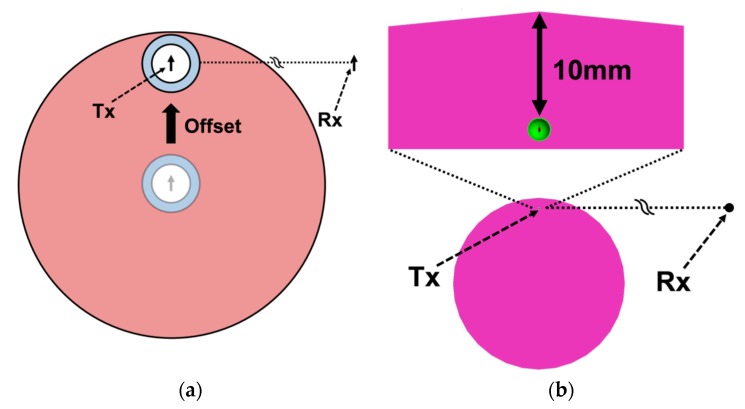
Off-center Tx model: (**a**) conceptual diagram; (**b**) model used for FEKO simulation.

**Figure A2:**
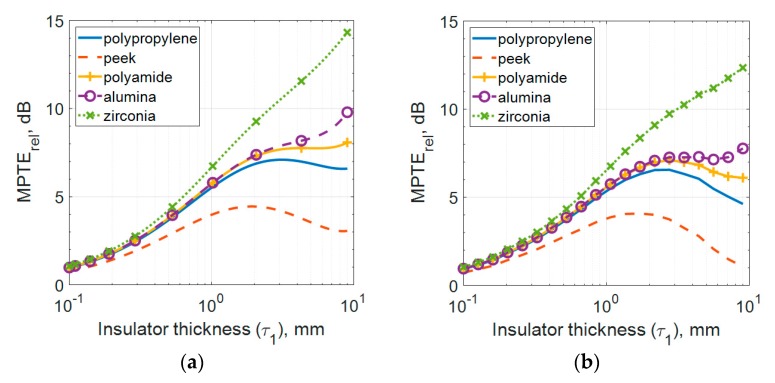
MPTE_rel_ results with commercially available biocompatible materials as an insulating layer at 2.4 GHz: (**a**) Figure 4b; (**b**) results of the off-center Tx model.

Whether or not the MSS model can accurately represent an antenna implanted in the head may be questioned, since the antenna is located at the center of the MSS, while it would be close to the top of the head in practice. To address this question and justify the model used here, we compare the calculations from our solution with numerical values when the Tx is located towards the top of the spherical model (Figure A1 and Figure A2). It can be observed that the overall tendency is largely matched with only a small difference of 1–2 dB.

## 3. Insulator Layer and Radiation Mode

In this section, we analyze the effect of the material properties and size of the insulator on MPTE using the derived closed-form expression to find the optimal configuration and radiation mode for implanted antennas. The frequencies of 403.5 MHz, 2.4 GHz and 5.8 GHz were chosen for this analysis. Table 1 presents the properties of commercially available biocompatible insulators and of the homogenous head layer in the MSS; the radius of the overall MSS (*r*_3_) was fixed at 9 cm [8,9,10,11,12,13]. Although the characteristics of the biocompatible materials in Table 1 could have been frequency dependent, they were set as constant in this study for convenience. That is because we focused on the generalized influence from dielectric properties, and the frequency dependence of real, specific material does not have impact on it.

Table 2 shows the calculated MPTE values using the derived solution for TM mode at 5.8 GHz when *r*_1_ and *τ*_1_ are both 1 mm and the insulator layer exhibits the dielectric constant and loss tangent of polyamide (i.e., *ε_r_* = 4.3 and *tanδ* = 0.004). The external monitoring system could be located either close to or far from the head. The results shown in Table 2 demonstrate that the absolute value of MPTE understandably varies depending on the distance to the Rx from the outer edge of the MSS (*r*-*r*_3_) and the position of the Rx with respect to the Tx (*θ*). To explore how much improvement might be made by changing the properties of the insulator, we then calculate the relative MPTE (MPTE_rel_) in comparison to an antenna without an insulating layer; that is, the MPTE of an MSS consisting of two layers of air and a homogenous head shell. As presented in Table 2, a clear improvement in MPTE of about 3.9 dB is observed for every case, with little difference between the various antenna distances and angles. In this preliminary study, the insulating layer was, therefore, found to improve MPTE regardless of the relative positions of the transmitting and receiving antennas. Subsequently, we fixed *r* = 5 m and *θ* = π/2, considering the situation that a patient and external monitoring system are far apart.

### 3.1. Effect of the Commercially Biocompatible Insulators

**Figure 4:**
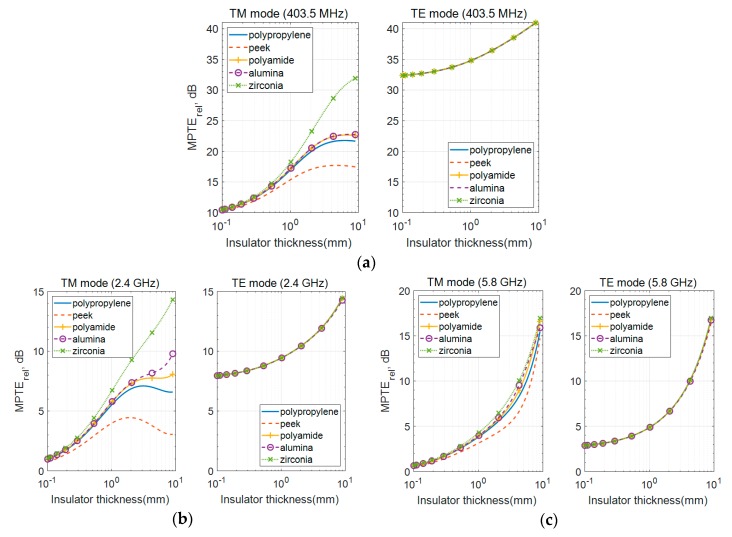
MPTE_rel_ results with different commercially available biocompatible materials as the insulating layer: (**a**) 403.5 MHz; (**b**) 2.4 GHz; (**c**) 5.8 GHz.

The MPTE_rel_ values according to *τ*_1_ thickness as it varies from 0.1 to 9 mm are shown in Figure 4 with *r*_1_ fixed at 1 mm. Although the insulator thicknesses up to 10 mm is not realistic for implanted antenna designs, such the long study range could show a clearer behavior of MPTE along the thickness. Both TM and TE modes are expressed in MPTE_rel_ with respect to the TM mode having no insulating layer. As shown in Figure 4, MPTE_rel_ for both TM and TE modes increases as the insulating layer becomes thicker in all frequency bands. In addition, MPTE_rel_ differs according to the kind of insulator used for the TM mode, where there is little difference for TE radiation, because the distribution of the magnetic near-field is less affected by the dielectric medium with no magnetic loss than that of the electric near-field.

Due to this characteristic, magnetic TE mode sources are known to be more efficient in power transfer than electric sources in a medium with dielectric loss, such as the human body [8,9,10,15]. However, as observed in Figure 4, the difference in MPTE_rel_ by insulator type also becomes smaller for TM radiation as frequency increases.

**Figure 5:**
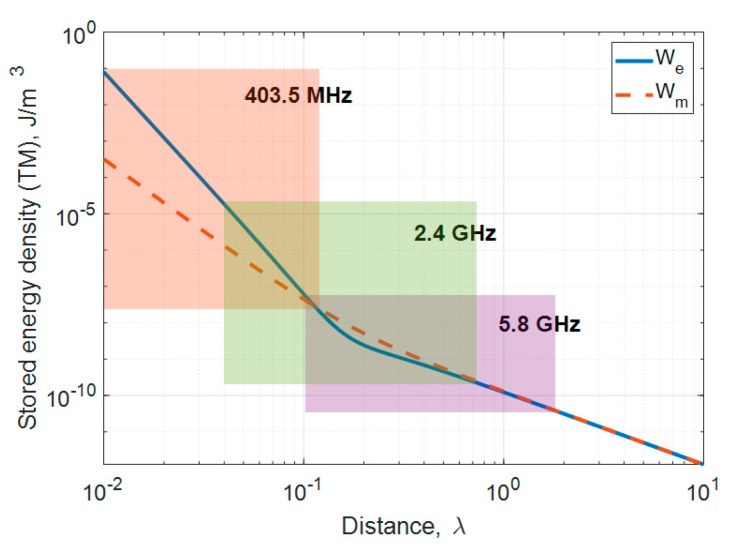
Stored electric and magnetic energy densities of a small dipole antenna.

The stored electric and magnetic energy densities of a small electric dipole according to distance in free space are plotted in Figure 5 to illustrate this phenomenon. The losses caused by MSS are divided into reactive near-field loss, propagating field absorption loss and reflection loss. The biggest influence on the difference between TM mode and TE mode loss is reactive near-field loss [10]. Here, it is shown that electric energy density (*w_e_*) is dominant closer to the source and that the difference between *w_e_* and the magnetic energy density (*w_m_*) becomes very small at greater distances. The colored areas represent the physical location of the insulating and head layers at each frequency band which occurs, for 403.5 MHz, with *w_e_* dominating, and, for 5.8 GHz, with the two density levels at almost the same value. Thus, the effect of the insulator is reduced at higher frequencies and the difference in MPTE_rel_ also decreases with small deviations between *w_e_* and *w_m_*. These findings from this sub-section can be summarized as follows:

- MPTE_rel_ is higher with thicker insulators.
- MPTE_rel_ of the TM mode varies according to insulator material but is almost negligible for TE radiation.
- As frequency increases, the effect on MPTE_rel_ by insulator type and the difference in both TM and TE mode MPTE_rel_ decreases.

### 3.2. The Effects of Variation in Dielectric Properties of Insulators

In Figure 4, it can also be observed that MPTE_rel_ is highest when zirconia, which has the lowest *tanδ* among the tested materials, is used and that MPTE_rel_ is lowest when peek, with the highest *tanδ*, is utilized. In contrast, alumina shows higher MPTE_rel_ than polypropylene and polyamide despite its *tanδ* being higher. In this section, MPTE_rel_ values are compared according to variations in the dielectric constant and loss tangent of the insulator, and this phenomenon is explained.

**Figure 6:**
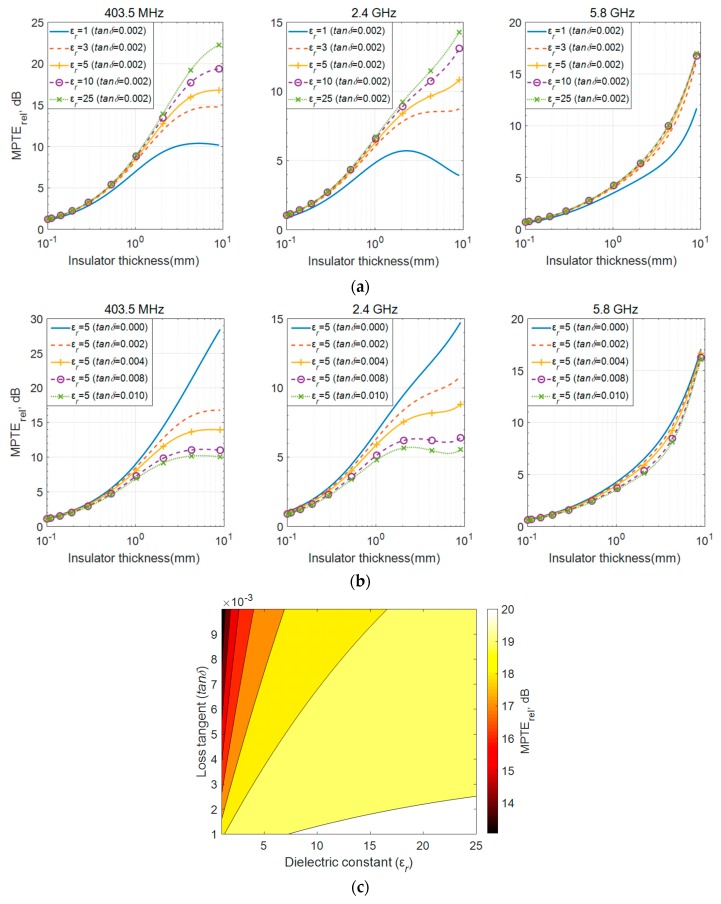
MPTE_rel_ results according to dielectric properties: (**a**) varying dielectric constant with stable loss tangent; (**b**) varying loss tangent with stable dielectric constant; (**c**) variations in *ε_r_* and *tanδ* at 403.5 MHz.

The MPTE_rel_ values for TM mode radiation according to these properties are plotted across *τ*_1_ in Figure 6. In Figure 6a, relative permittivity is adjusted while loss tangent is maintained, and in Figure 6b, loss tangent varied while keeping relative permittivity constant. The properties of the head layer still follow those in Table 1. The figures show that higher MPTE_rel_ values are obtained as the dielectric constant of the insulator increases and the loss decreases. This explains the high MPTE_rel_ of zirconia (Figure 4), which has the highest dielectric constant and the lowest loss tangent among the tested materials. When comparing polypropylene, polyamide and alumina in terms of MPTE_rel_ (see Figure 4), it is found that alumina, with its higher loss tangent and higher dielectric constant, shows a higher MPTE_rel_ than the other two materials. For polypropylene and peek, also as shown in Figure 4, peek demonstrates a lower MPTE_rel_ due to its loss being three times higher, although its dielectric constant is only marginally higher.

We plotted MPTE_rel_ against the variations in dielectric constant and loss tangent at 403.5 MHz in Figure 6c to explore which configuration has the greatest impact on MPTE_rel_. Therein, *τ*_1_ is fixed at 1 mm because practical insulator thickness is usually around 1 mm [4,5,6,7]. It is interesting to observe that there is significant variation in MPTE_rel_ values as *tanδ* changes when *ε_r_* is less than 5.0, as with polypropylene, peek and polyamide. In contrast, MPTE_rel_ is less affected by relatively large *tanδ* values when *ε_r_* is greater than 5.0, as with alumina and zirconia. This explains why alumina, with a higher *tanδ*, shows better MPTE_rel_ than polypropylene and polyamide. Similar trends are observed for higher frequencies, although the effect is not as pronounced as with 403.5 MHz. This can be inferred from the case of 5.8 GHz in Figure 6a,b where the differences between the MPTE_rel_ values are not huge.

From the findings presented in this section, it can be said that improvement in MPTE_rel_ is not significant when the dielectric constant is greater than 5.0 with an insulator thickness of 1 mm. Materials with a high dielectric constant and low loss are rare and usually expensive, but these findings indicate that materials with a dielectric constant greater than 5.0 are sufficient for selection as the insulating layer, since they will be much less affected from the higher loss tangent.

### 3.3. Lossless Air Region

In the MSS model, we placed the Tx in a lossless air region to address the limitations of the small antenna being located in a lossy medium. It was shown in the previous section that MPTE_rel_ can be improved with the thicker thickness and higher dielectric constant of the insulating layer. Such a MPTE_rel_ improvement is also possible by the increased size of the lossless region [10]. The implanted antenna may be insulated in one of two ways: One is to coat the antenna directly with the insulating material [7], and the other is to place the antenna in a structure such as a capsule, which works as an insulator [4].

**Figure 7:**
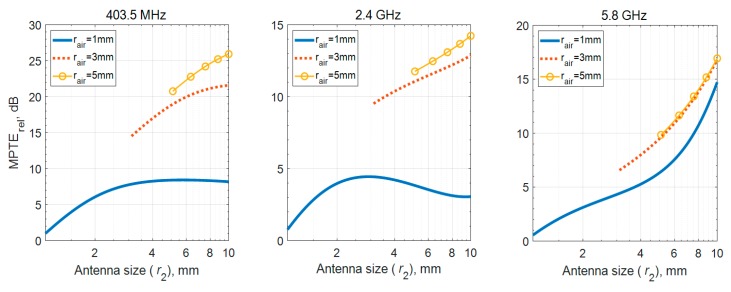
MPTE_rel_ results according to antenna size adjusted by air region.

The biggest difference between these two insulating methods is the presence or absence of the lossless area. To analyze the effect of the lossless air region, the MPTE_rel_ values are compared according to different *r*_1_ sizes with the same antenna. Antenna size (*r*_2_) combines the thickness of the insulating layer and the radius of the air region. To examine the effect on the MPTE_rel_ from the lossless air region clearer, the peek with the lowest MPTE_rel_ is chosen as the insulator material. Figure 7 shows the MPTE_rel_ according to *r*_2_. It is shown that the higher MPTE_rel_ values are obtained at the same *r_2_* when the lossless air region is larger, meaning that having more lossless region could be more suitable for increasing MPTE_rel_ than using thick insulating layer, as observed from Figure 7. From a practical point of a view, using a capsule to secure the air region should inevitably increase the size, but it can also increase the MPTE_rel_ of the antenna immersed in the lossy medium. If a thin and rigid cover could be built for a planar implanted antenna, it not only improve antenna radiation properties but also work as insulation.

### 3.4. Radiation Mode

**Figure 8:**
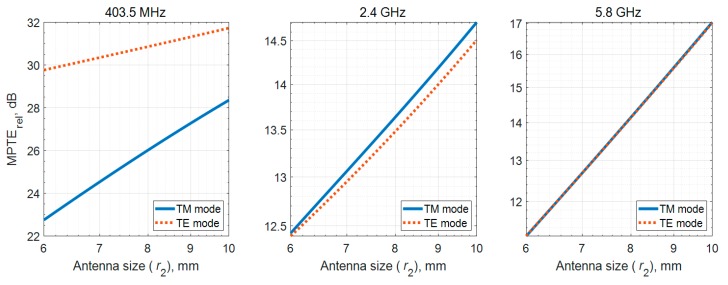
MPTE_rel_ results when the insulator layer is composed of zirconia, which is the optimal material based on previous results.

As previously outlined, it is known that a magnetic source in the human body is less affected than an electrical source. However, as shown in Section 3.1, the difference between TM and TE radiation modes decreases as frequency increases for a model with a specific physical size. In addition, the upper bound of radiation efficiency for a TE_10_ mode dipole has been found to be limited [23]. Thus, we compare MPTE_rel_ for the different radiation modes using zirconia, which has the highest dielectric constant and lowest loss, and a fixed *r*_1_ of 5 mm. As shown in Figure 8, the MPTE_rel_ of the TE mode appeared several dB higher than TM at 403.5 MHz, whereas no big differences were observed at 2.4 GHz or 5.8 GHz. These results assume 100% radiation efficiency. However, antennas made of metals with finite conductivity such as copper, should present an upper limit to radiation efficiency obtainable from the finite electrical antenna size. When the antenna is electrically small, the radiation efficiency of the TE mode is significantly lower than that of the TM mode. For example, whereas TE mode radiators could be designed to have radiation efficiency close to 100% at 2.4 GHz and 5.8 GHz with 10 mm of the maximum dimension of the antenna, but at 403.5 MHz only 4.4%, according to [23]. An implanted antenna at 403.5 MHz must be very small and thin which will affect MPTE_rel_.

Furthermore, an electrically small antenna has a minimum *Q* bound depending on its size, and the *Q* value of a TE mode antenna is twice as high as a TM mode antenna of the same size [24]. In order to overcome the narrow bandwidth limitation of such antennas, they are designed either in a relatively complex three-dimensional configuration [25,26,27] or with an active circuit such as a non-Foster element [28,29]. Thus, considering actual antenna design, TM mode may be more suitable across each frequency band.

## 4. Conclusions

In this study, the influence of the insulating layer of an implanted transmitting antenna on the relative maximum power transfer efficiency to an external receiving antenna was analyzed through a derived analytic solution. Theoretical MPTE values were obtained using an MSS model which separates the Tx and Rx antennas using spherical wave theory. The derived expression was validated through the full-wave EM simulator FEKO. The effects of the insulating layer on MPTE_rel_ at 403.5 MHz, 2.4 GHz and 5.8 GHz were analyzed using variance in dielectric properties, different insulator sizes, and possible radiation modes.

The insulating material has very small effects on TE mode radiation, but there is a meaningful difference in MPTE_rel_ in the case of the TM mode. In TM mode, it was shown that a higher dielectric constant can produce a higher MPTE_rel_ value. This property holds true even for a material with a higher loss tangent if its dielectric constant is greater than 5.0. It was also shown that the loss tangent does not have a significant impact on MPTE_rel_ when the dielectric constant is very high. However, for low dielectric constants, the effects of the surrounding lossy material become significant. This general behavior explains the different MPTE_rel_ values derived from commercially available biocompatible insulating materials. Next, the proper layout of the insulator was discussed as inferred from the size of the air region around the Tx in the MSS model. It was shown that MPTE_rel_ was improved when the lossless air region increased. It will be challenging to create such an antenna within a thin and rigid cover, but using the novel design methods of 3D printing technologies, we hope to present further results imminently. Finally, it was shown that MPTE_rel_ differed by radiation mode. For perfect radiation efficiency, the TE mode shows higher MPTE_rel_ at 403.5 MHz, whereas the values are approximately equal with TM radiation for the higher frequencies. When taking into account the limited upper radiation efficiency bound of a small TE mode antenna, however, the achievable MPTE_rel_ becomes significantly lower, leading to the conclusion that a small TM_10_ antenna would be more effective across all possible medical bands in terms of power transfer efficiency. Overall, optimum insulator design and radiation mode were thoroughly investigated, and the results of this study represent robust design guidelines for increasing the reliability of wireless biotelemetry systems involving brain-implanted antennas.

## Figures and Tables

**Table 1:** Material properties of biocompatible insulators and the homogenous head layer.

Material	Dielectric Constant (*ε_r_*)	Loss Tangent (*tanδ*)
Polypropylene *	2.55	0.003
Peek *	3.20	0.010
Polyamide *	4.30	0.004
Alumina *	9.20	0.008
Zirconia *	29.0	0.002
Head tissue (400 MHz)	43.5	0.799
Head tissue (2.4 GHz)	39.2	0.344
Head tissue (5.8 GHz)	35.3	0.463

* biocompatible material. Note: the biocompatible materials are dependent on frequency but were set to be frequency independent in this study.

**Table 2:** Absolute and relative MPTE results by distance and angle between Tx and Rx when insulator is polyamide at 5.8 GHz.

	MPTE (Absolute)	MPTE (w/o Insulator)	Improved MPTE (Relative)
*r* − *r*_3_ = 1 cm and *θ =* 0	1.11 × 10^−17^	4.51 × 10^−18^	3.911 dB
*r* − *r*_3_ = 1 cm, and *θ =* π/2	1.64 × 10^−24^	6.67 × 10^−25^	3.907 dB
*r* − *r*_3_ = 5 m, and *θ =* 0	4.01 × 10^−16^	1.63 × 10^−16^	3.910 dB
*r* −*r*_3_ = 5 m, and *θ =* π/2	1.57 × 10^−19^	6.38 × 10^−20^	3.911 dB

## Appendix A

In Section 3, the insulating layer of the implanted antenna was analyzed using an MSS model. The biggest limitation of our MSS is that the Tx is at the center of the shell where it would be located near the top of the head in practice. In order to demonstrate that the conclusions using the derived solution are applicable to an actual implanted antenna, we compare an off-center Tx simulation where the antenna is not centered but close to the edge of the shell in Figure A1.

Figure A1a is a conceptual diagram of the Tx off-center by a certain distance, and the actual simulation model is shown in Figure A1b. The *r*_1_ is fixed at 1 mm and moved so that the outermost edge of the antenna is 10 mm away from the top edge of the outer layer. The Rx is in parallel with Tx and is 5 m away. An electric dipole measuring λ/125 in length with a radius of 1 mm is used. Figure A2a repeats Figure 4b, and Figure A2b compares the simulation results of Figure A1b under the same conditions. As can be seen, the MPTE_rel_ values of either case deviate by just 1–2 dB, and, importantly, the tendency is well maintained.

According to [9,30] the radiating power of the implanted antenna is higher when the TM or TE dipole stands parallel to the shell edge and when the source antenna is located closer to the shell edge. In other words, MPTE_rel_ is dependent on both the polarization of the electric field joining the MSS edge and the variation of the propagation path. The MPTE_rel_ decreases for the off-set case in Figure A2b compared to Figure A2a despite that the Tx is located closer to the edge (see. Figure A1b), because Tx dipole is no more parallel to the shell edge but is very tilted. The fact that the insulating layer mostly affects the mismatch at the adjacent boundary of the head layer [8] can explain the same trend of the graphs in Figure A2a,b.

## Appendix B

A list of acronyms and abbreviations is given in Table A1 for the convenience of the readers.

**Table A1:** List of acronyms and abbreviations.

Acronym (Abbreviation)	Definition
MPTE	maximum power transfer efficiency
MICS	medical implant communication system
ISM	industrial, scientific and medical
UWB	ultra-wideband
MSS	multi-layered spherical shell
Tx	transmitting antenna
Rx	receiving antenna
MPTE_rel_	relative maximum power transfer efficiency
